# Vaccine-Induced Adverse Effects in Cultured Neuroblastoma 2A (N2A) Cells Duplicate Toxicity of Serum from Patients with Gulf War Illness (GWI) and Are Prevented in the Presence of Specific Anti-Vaccine Antibodies

**DOI:** 10.3390/vaccines8020232

**Published:** 2020-05-18

**Authors:** Effie-Photini C. Tsilibary, Eric P. Souto, Marian Kratzke, Lisa M. James, Brian E. Engdahl, Apostolos P. Georgopoulos

**Affiliations:** 1 Brain Sciences Center, Department of Veterans Affairs Health Care System, Minneapolis, MN 55417, USA; tsili001@umn.edu (E.-P.C.T.); souto014@umn.edu (E.P.S.); kratz031@umn.edu (M.K.); lmjames@umn.edu (L.M.J.); engda002@umn.edu (B.E.E.); 2 Department of Neuroscience, University of Minnesota Medical School, Minneapolis, MN 55455, USA; 3 Department of Psychiatry, University of Minnesota Medical School, Minneapolis, MN 55454, USA; 4 Department of Psychology, University of Minnesota, Minneapolis, MN 55455, USA; 5 Department of Neurology, University of Minnesota Medical School, Minneapolis, MN 55455, USA

**Keywords:** Gulf War illness, N2A cultures, apoptosis, vaccines, anti-vaccine antibodies

## Abstract

Gulf War illness (GWI) is a chronic disease of unknown etiology affecting over 200,000 veterans with symptoms including neurocognitive problems. We previously demonstrated GWI serum toxicity on neural cell cultures manifested by compromised neural network function, decreased cell spreading, and enhanced cell apoptosis. These patients lacked six human leukocyte antigen (HLA) class II alleles, resulting in an inability to form antibodies. Therefore, we hypothesized that GWI patients have vaccine-derived, persistent pathogens, which contribute to the development of the disease. Here, we examined whether individual vaccines were toxic in cultured N2A cells. Moreover, we used antibodies against each of the 20 vaccines administered to Gulf War (GW) veterans, to examine the effects of these antibodies on cell spreading and apoptosis in N2A cells. Antibodies against cholera toxin, hepatitis B, hemagglutinin H1N1, H3N2, and B from influenza A and B strains, measles, and *Salmonella Typhi* polysaccharide Vi had a remarkable protective effect on both cell spreading and apoptosis, whereas none of the other antibodies administered to GW veterans had an effect. The in vitro observed adverse effects of GWI serum may be due in part to vaccine-derived pathogens, antibodies against which had a protective effect in N2A cell cultures.

## 1. Introduction

After the Persian Gulf War of 1990–1991, about one-third (>200,000) of deployed veterans complained of a variety of chronic physical and neurocognitive symptoms [1,2,3,4], which are presently identified as Gulf War illness (GWI). We previously described a number of functional and structural brain abnormalities in GWI, such as changes in synchronous neural communication patterns [5], and the presence of subcortical brain atrophy in certain GWI patients [6]. This atrophy was absent in veterans carrying the human leukocyte antigen (HLA) allele DRB1*13:02 [7], one of six HLA class 2 alleles that we reported previously as protective for GWI [8]. Moreover, with fewer of those alleles carried, the symptomatology became more severe. The function of HLA class 2 alleles is specific immunity, through which external antigens are presented to CD4^+^ lymphocytes, leading ultimately to the production of specific antibodies by B cells to neutralize the offending antigen [9]. Given these considerations, we hypothesized that the lack of HLA class II protection observed in GWI would have allowed offending antigens to persist.

Long-lasting persistence of administered antigens following immunization was reported [10,11]; therefore, it is feasible that one or several antigens/pathogens also are present in GWI patients, leading to our “persistent antigen” hypothesis [7]. We hypothesized that such persisting antigens could be derived from vaccines administered to GW veterans, possibly leading to cell damage and low-grade inflammation, as well as contributing to the multi-symptom chronic GWI. According to this hypothesis, healthy GW veterans carrying protective alleles would have specific antibodies in their blood, which could neutralize the hypothesized persistent antigens present in GWI serum. In order to test this prediction, (a) we assessed the effect of GWI serum on function and morphology of neural cultures in vitro, and (b) we tested veterans [12]. Indeed, we found that (a) GWI serum exerted harmful effects on neural cultures, and (b) those effects were prevented by the addition of serum from healthy Gulf War (GW)-era veterans. These findings suggest that healthy serum may contain antibodies against harmful antigens present in GWI serum; if so, such antibodies may hold promise for a successful intervention in treating GWI. We initially tested the possible beneficial effect of pooled human antibodies by adding to the culture pooled human immunoglobulin G (IgG) in N2A cultures simultaneously with GWI serum. Human IgG should contain antibodies against a broad range of common pathogens partially overlapping with those contained in the vaccines administered to GW veterans. Indeed, we found a highly significant and substantial beneficial effect [13]. We next addressed the specific effect of antibodies against anthrax, a rare antigen administered as the anthrax vaccine (“Biothrax”) to GW veterans, by co-incubating anti-anthrax antibodies with GWI, which was then added to N2A cultures, and we observed a remarkable protective effect [14]. Our previous data then suggest the presence of vaccine-derived pathogens in GWI patients. In the present report, we examined the following: (a) whether individual vaccines were toxic in cultured N2A cells, (b) whether GWI serum toxicity duplicated toxic effects of GW-era vaccines, and (c) whether antibodies against each of the 20 vaccines administered to GW veterans had protective effects on N2A cell spreading and apoptosis.

## 2. Materials and Methods

### 2.1. Serum

Serum from 15 GWI veterans with substantial neurocognitive symptoms and no protective alleles [11], in addition to serum from one healthy GW-era control veteran, who was free of neurocognitive symptoms and had two of the six HLA protective alleles, was used. For all participants, informed consent was obtained prior to the experiment and all procedures were approved by the relevant Institutional Review Boards.

### 2.2. Cell Culture

#### 2.2.1. Vaccines

Neuro-2A neuroblastoma cells were cultured in Eagle’s minimal essential medium (EMEM, ATCC, VA, USA) containing 10% fetal bovine serum (ThermoFisher Scientific, Waltham, MA, USA) and 1% penicillin–streptomycin (ThermoFisher Scientific, Waltham, MA, USA). In sequence, cells were seeded in poly-d-lysine-coated, 24-well plates at a concentration of 100,000 cells/well in Neurobasal medium containing N2 supplement and l-glutamine (ThermoFisher Scientific, Waltham, MA, USA), in the absence (medium control) or presence of human serum. Then, 48 h later, 10–40 μL of the following vaccines were added: influenza (fluarix quadrivalent, containing A/California/7/2009 (H1N1)-like virus, A/Switzerland/9715293/2013 (H3N2)-like virus B/Phuket/3073/2013-like virus, GlaXoSmithKLine, Philadelphia, PA, USA [15]); typhoid (TYPHIM-Vi SanofiPasteur, Bridgewater, NJ, USA [16]); rabies (IMOVAX, SanofiPasteur, Bridgewater, NJ, USA [17]); meningococcus containing groups Ac,C,Y and W-135 (Menactra, SanofiPasteur, Bridgewater, NJ, USA [18]); MMR (measles, mumps, rubella, M-M-R-II (SanofiPasteur, Bridgewater, NJ, USA [19]), varicella (Varivax, Merck Vaccines, West Point, PA, USA [20]); polio (IPOL, SanofiPasteur, Bridgewater, NJ, USA [21]). Vaccines containing aluminum (hepatitis A and B, Japanese encephalitis, tetanus, diphtheria, etc.) were all toxic to cells and, after a test experiment, were not used further, whereas others, including cholera and yellow fever vaccines, were not available.

The list of vaccines administered to Gulf War veterans included the following vaccines inactivated (or attenuated) pathogens: adenovirus (types 4 and 7), anthrax, botulinum toxoid, cholera, hepatitis B, influenza, measles, meningococcus (A,C,Y,W135), mumps, plague, polio, rabies, rubella, smallpox, tetanus-diphtheria, typhoid, varicella, and yellow fever.

Dosage of vaccines added per 1 mL of medium in each culture were as follows: influenza, 0.9, 1.8, 2.7, and 3.6 μg; meningococcus, 0.3–0.6 μg; rabies, 0.0125–0.025 units of rabies antigen; MMR, 20–30 TCID50 (tissue culture infectious doses) of measles virus 27–54 pfu (plaque forming units); polio, 0.8- and 1.6-D antigen units of Type 1,8 D and 0.64- and 1.28-D antigen units of type 3 poliovirus *Salmonella Typhi*: 0.5–1 μg of purified Vi polysaccharide.

Detailed information about these vaccines is described on their respective websites [15,16,17,18,19,20,21].

In preliminary experiments, cells transferred from EMEM plus 10% FBS to Neurobasal medium were examined for spreading at 1, 2, 3, 5, and 6 days; the optimal time was observed to be two days (48 h), when control cells (cultured in medium) were spread extensively. On the first day, cell spreading was minimal, whereas at 3, 5, and 6 days, control cells were overgrown and hard to examine. Therefore, exposure of Neuro 2A cells to treatment in all experiments was set to 48 h, when the cells were examined. Five to 10 fields were obtained from a phase-contrast microscope Motic AE2000-Trinocular inverted microscope (Ted Pella, Redding, Ca, USA), with a Zeiss Axiocam 105 color digital camera (Carl Zeiss Microscopy, LLC, Thornwood, NY, USA). 

#### 2.2.2. Antibodies

Neuro-2A neuroblastoma (N2A) cells were cultured in Eagle’s minimal essential medium (EMEM, ATCC, VA, USA) containing 10% fetal bovine serum (ThermoFisher Scientific, Waltham, MA, USA) poly-d-lysine coated, 24-well plates at a concentration of 30,000–50,000 cels/well for 48–72 h. The medium was then changed to Neurobasal containing N2 supplement and l-glutamine (ThermoFisher Scientific, Waltham, MA, USA), in the absence (medium control) or presence of human serum for another 24–48 h; then, the medium was changed and cells were exposed to treatment for a total of 48 h. In these experiments, human serum was added in three combinations: control (10%), GWI (10%), plus antibodies (5–10 μg each).

For all experiments, at day 2 post-treatment, the cells were examined with a phase-contrast microscope and 5–10 fields of each differently treated culture were photographed. The photographs were examined for the presence of aggregated cells and cells with or without processes.

### 2.3. Cell Morphology Assay—Process Formation

The effect of antibodies to GW-era vaccines on the morphology of N2A cells was examined. For this assay, the following antibodies were each incubated with GWI serum: anti-hemagglutinin (HA)–H1N1 (Cat. No. 11055-RP01), anti-HA–H3N2 (Cat. No. 11715-RP01), anti-HA–B (Cat. No.11053-RP01), strains of influenza (polyclonal, Sino Biological, Wayne, PA, USA); anti-*Salmonella* (Cat. No. PA1-20811, polyclonal, ThermoFisher Scientific, Rockford, IL, USA); anti-Japanese encephalitis virus (Cat. No. PA5-32237, polyclonal, ThermoFisher Scientific, Rockford, IL, USA); anti-*Yersinia pestis* (Cat. No. MA1-23074, monoclonal, ThermoFisher Scientific, Rockford, IL, USA); anti-hepatitis virus B (Cat. No. ab9193, polyclonal, Abcam, Cambridge, UK); anti-cholera toxin (monoclonal, Cat. No. LS-C142039, LS Bio, Seattle, WA, USA); anti-Yellow fever (Cat. No.3576, ThermoFisher Scientific, Rockford, IL, USA); anti-varicella zoster (Cat. No.LS-132860/58089, LS Bio, Seattle, WA, USA); anti-anthrax protective antigen (Cat. No. CPBT-66806RA, polyclonal, Creative Diagnostics, Shirley, NJ, USA); anti-mumps virus (Cat. No. MBS320375, monoclonal, MyBioSource, San Diego, CA, USA); anti-adenovirus (Cat. No. LS-C63691/120157, Seattle, WA, USA); anti-polio virus (Cat. No. ab22450, monoclonal, abcam, Cambridge, UK); anti-clostridium botulinum B toxoid activity (polyclonal, Cat. No. ab83064, abcam, Cambridge, UK); anti-measles (monoclonal, Cat. No. DMABT-H21849 Creative Diagnosticss, Shirley, NJ, USA). Antibodies were titrated for effects at a series of concentrations following pre-incubation with GWI serum and used at the lowest active concentration of 5–10 μg/mL each. Each separate antibody and a combination of all anti-influenza HA antibodies were incubated at 10 μg each with 100 μL of GWI serum for 60 min at 37 °C, and then added in a final volume of 1 mL of Neurobasal medium containing N2 supplement and l-glutamine. 

The N2A cells were cultured with GWI serum pre-incubated in the presence or absence of each of these antibodies for two more days and the cells were photographed. Images were obtained from 5–8 different fields per sample, from a minimum of three experiments using a Motic AE2000-Trinocular inverted microscope (Ted Pella, Redding, CA, USA), with a Zeiss Axiocam 105 color digital camera (Carl Zeiss Microscopy, LLC, Thornwood, NY, USA). The extent of cell spreading was then calculated with ImageJ software by measuring the number of cells with processes relative to the total cell number.

### 2.4. Cell Apoptosis Determined with Terminal Deoxynucleotidyl Transferase-Mediated dUTP Nick End Labeling Assay (TUNEL) Assay

The extent of cell apoptosis of Neuro-2A cells was examined at two days post-treatment with vaccines or antibodies, using four- and eight-chamber glass slides (ThermoFisher Scientific, Waltham, MA, USA) coated with poly-d-lysine at 50 μg/mL as mentioned above. N2A cells were seeded at a concentration of 50,000–100,000 cells per chamber, in 1 mL of Neurobasal/N2/l-glutamine medium for two days. In sequence, 10% of GWI serum, incubated for 60 min at 37 °C in the presence or absence of 5–10 μg of each of the above-mentioned antibodies, and in addition to 10 μg/mL of H1N1, H3N2, and B strains of influenza hemagglutinin each, was added for two more days. At the end of the incubation period, the cells were examined for apoptosis. Apoptotic cells were detected using the In Situ Cell Death Detection Kit, TMR red (terminal deoxynucleotidyl transferase (TdT) enzyme and fluorochrome labeling solution), according to the manufacturer’s protocol. Briefly, the cells were fixed in ice-cold methanol for 10 min at room temperature, rinsed with phosphate-buffered saline (PBS), and permeabilized with 0.1% Triton X-100 in PBS for 3 min on ice. The cells were then incubated with 150 μL of TUNEL reaction mixture for 60 min at 37 °C in the dark (In situ Cell Death Detection Kit, TMR red, ThermoFisher scientific, Waltham, MA, USA). The cells were washed 3× with PBS and Diamond AntiFade mounting medium with 4’,6-diamidino-2-phenylindole (DAPI) stain (ThermoFisher Scientific, Waltham, MA, USA) used for visualization of nuclei, using the EVOS FL Cell Imaging System (ThermoFisher Scientific, Waltham, MA, USA). Eight to 10 images were obtained from different fields from a minimum of two experiments with each different experimental condition. Apoptosis was then calculated with ImageJ software by measuring the number of TUNEL-labeled cells (red nuclei) relative to the total cell number (DAPI-stained nuclei).

### 2.5. Statistical Analyses

N2A cells treated with GWI serum, vaccines, and/or antibodies were tested as to whether they had different/altered apoptosis/spreading compared to controls (healthy serum, plain media, dependent on the experiments). Since experiments were run concurrently, pairwise comparisons of antibody (and/or vaccine) vs. the relevant control (healthy serum or plain media, depending on the experiment) were made using the paired *t*-test of the Microsoft Excel program.

### 2.6. HLA Genotyping

DNA isolation was carried out from 3 mL of whole blood drawn in ethylenediaminetetraacetic acid (EDTA) tubes, using a commercially available kit (ArchivePure cat. 2300730) from 5Prime (distributed by Fisher Scientific or VWR) with an expected yield of 50–150 μg of DNA. The purified DNA samples were sent to Histogenetics [22] for high-resolution HLA sequence-based typing [23,24] 

Their sequencing DNA templates were produced by locus- and group-specific amplifications that include exon 2 and 3 for class I (A,B,C) and exon 2 for class II (DRB1, DRB3/4/5, DQB1, and DPB1) and reported as antigen recognition site (ARS) alleles as per ASHI recommendation14. All 15 GWI participants had neurocognitive/mood symptoms and lacked any of the six HLA alleles protective for GWI (DRB1*01:01, DRB1*08:11, DRB1*13:02, DQB1*02:02, DPB1*01:01, DRB1*06:01) [3]. The control participant was homozygous for the DRB1*01:01 protective allele.

## 3. Results

### Vaccines Used in N2A Cell Cultures 

**Figure 1:**
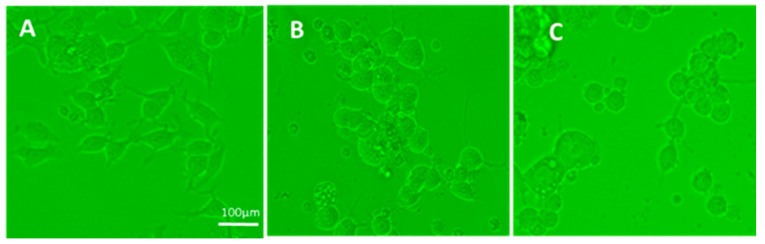
(**A**) Control N2A cells: cells spread and formed processes as early as two days in culture in the presence of medium only; (**B**) N2A cells two days after exposure to influenza vaccine; (**C**) N2A cells two days after exposure to *S. Typhi* vaccine. In both B and C, cells were mostly rounded and deprived of processes (either could not form and/or retracted processes), compared to cells cultured in the presence of medium.

**Figure 2:**
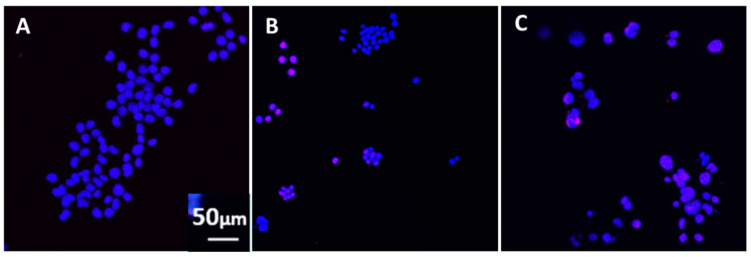
(**A**) Control (blue, 4′,6-diamidino-2-phenylindole (DAPI)) staining of N2A cell nuclei indicates healthy cells. The cells were cultured in the absence (**A**) or presence (**B**) of influenza vaccine or (**C**) *S. Typhi* vaccine for two days. Red-stained, terminal deoxynucleotidyl transferase-mediated dUTP nick end labeling (TUNEL) nuclei indicate DNA breaks and, hence, apoptotic cells. DAPI (blue)-stained nuclei represent nuclei of healthy cells.

**Figure 3:**
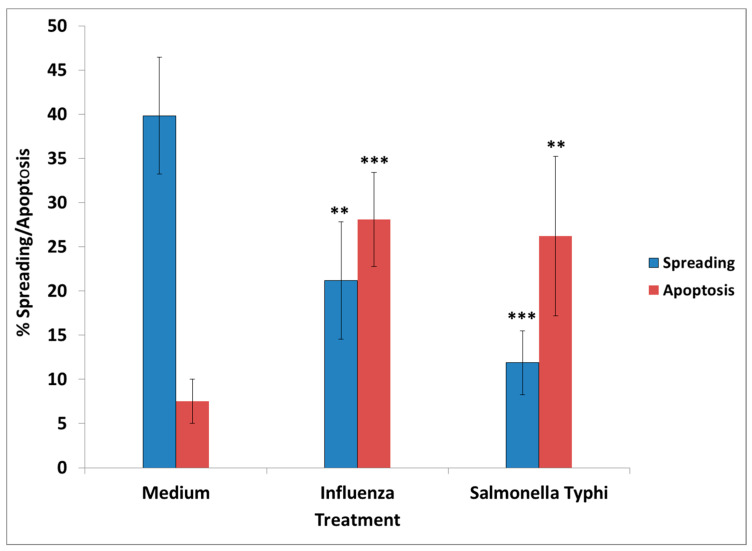
Percentage spreading and apoptosis of N2A in the presence of influenza and *S. Typhi* vaccine. ** p < 0.001, *** p < 0.0001 (compared to the medium). Spreading was decreased >15% by influenza and >25% by *S. Typhi*; apoptosis was increased >25% by influenza and >20% by *S. Typhi*.

The following vaccines tested had an adverse effect when added in N2A cultures, causing cells to aggregate and detract/not form processes: cholera toxin, hepatitis B, hemagglutinin H1N1, H3N2, and B from influenza A and B strains, measles, and *S. Typhi* polysaccharide Vi. Two examples are shown in Figure 1. Significantly decreased cell spreading with lack of processes, compared to the control (medium), was observed in cells exposed to the influenza (~20% decrease) and the *S. Typhi* vaccine (~30% decrease), at two days post-vaccine exposure. Apoptosis was increased by >3× compared to the control (Figure 2). Moreover, there was significant apoptosis observed in the presence of “toxic vaccines” added to N2A cultures, as shown in Figure 2. Percentage spreading and apoptosis of N2A in the presence of influenza and *S. Typhi* vaccine are shown in Figure 3. Spreading was decreased >15% by influenza and >25% by *S. Typhi*; apoptosis was increased >25% by influenza and >20% by *S. Typhi*.

**Figure 4:**
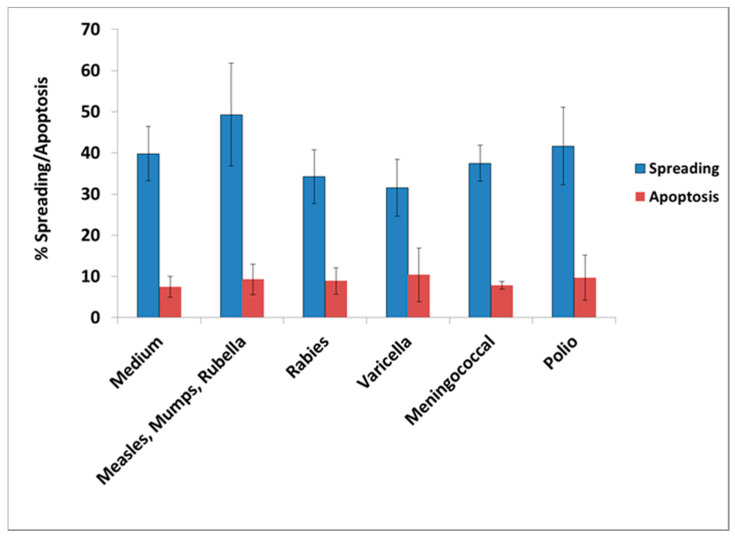
Percentage cell spreading and apoptosis in N2A cells cultured for 48 h in the absence (medium) or presence of each of the vaccines: MMR (measles, mumps, rubella), rabies, varicella, *Neisseria meningitis*, and polio virus. These vaccines did not have a significant effect on cell spreading or apoptosis of N2A cells compared to the control (medium) by the paired *t*-test.

Most of the other tested vaccines did not have a significant effect on the extent of cell spreading and apoptosis. The following vaccines were tested: Polio, varicella, meningococcus, and measles–mumps–rubella, as shown in Figure 4.

**Figure 5:**
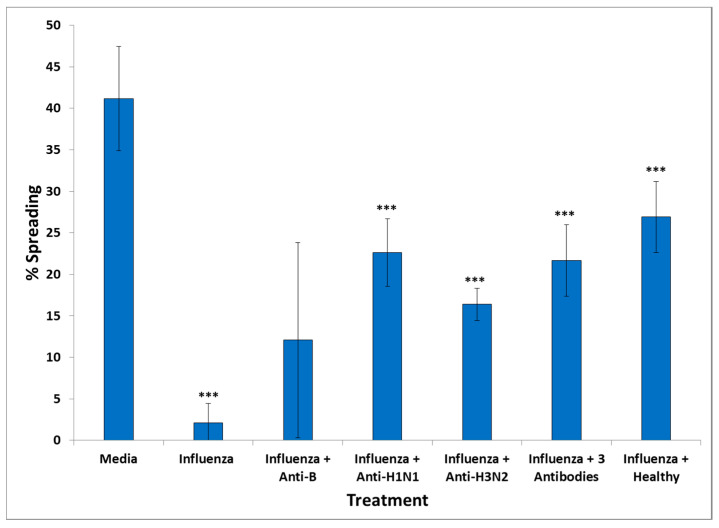
Effect of influenza vaccine, and influenza vaccine incubated with antibodies to influenza vaccine components, H1N1, H3N2, and B strains or without the influenza vaccines on N2A cell spreading. *** *p* < 0.0001 (compared to medium).

To confirm that the observed effect of harmful vaccines was specifically due to the pathogen they contained, we used antibodies against each of the three antigens contained in the influenza vaccine which were hemagglutinin H1N1, hemagglutinin H3N2 (strain A of influenza), and hemagglutinin B (strain B). Antibodies against each of these hemagglutinins were co-incubated with the influenza vaccine at 5 μg/mL with 15 μL of the vaccine for 1 h at 37 °C and then added to N2A cells for 48 h, before assessing cell spreading. We observed that influenza decreased cell spreading by almost 8×. Antibodies against influenza B hemagglutinin did not have a significant effect, but antibodies to H1N1 hemagglutinin increased cell spreading by almost 5× and antibodies to H3N2 increased cell spreading by 4×. A combination of the three antibodies also increased N2A cell spreading by almost 5× (Figure 5). 

**Figure 6:**
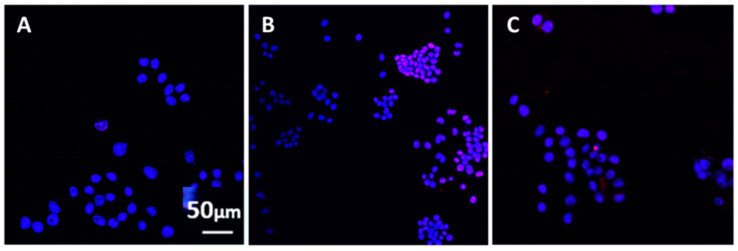
Ν2A cell apoptosis of cells in medium (**A**), exposed to the influenza vaccine (**B**) and (**C**) in the simultaneous presence of influenza vaccine and antibodies against H1N1. Similar effects were observed when antibodies to H3N2, and *S. Typhi* were used. The antibodies were added to the culture medium simultaneously with the corresponding vaccine for two days. In the presence of the influenza vaccine, most nuclei were stained red with TUNEL indicating apoptosis (B), whereas, in the combined presence of the influenza vaccine and anti-H1N1 antibodies (C), most nuclei appeared healthy and stained with DAPI, similar to control cells cultured in medium (A).

Antibodies against H1N1 also protected against influenza-induced cell apoptosis since most nuclei appeared healthy with the TUNEL assay (Figure 6).

Apparently, components of the influenza strain had a significant toxic effect on N2A cells, as indicated by decreased spreading and apoptosis. Notably, this effect could be partly prevented by pre-incubating the influenza vaccine with antibodies anti-influenza antibodies.

#### Effects of Vaccine Antibodies Co-Incubated in the Presence of GWI Serum

**Figure 7:**
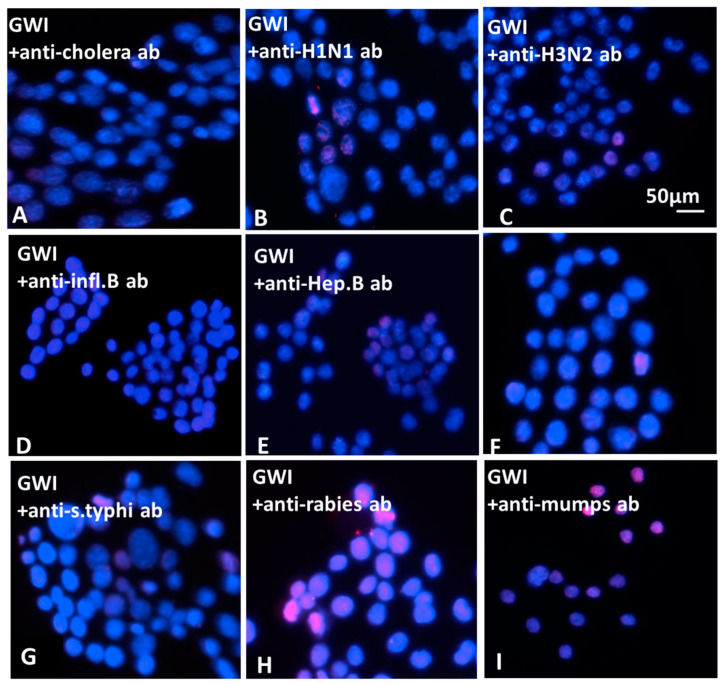
Ν2A cell apoptosis of cells in the presence of Gulf War illness (GWI) plus antibodies to cholera (**A**), influenza A/H1N1 (**B**), influenza A/H3N2 (**C**) influenza B (**D**), hepatitis B (**E**), measles (**F**), *S. Typhi* (**G**), rabies (**H**), and mumps (**I**). The antibodies were added in the culture medium simultaneously with GWI serum for two days. Most nuclei appeared healthy and stained with DAPI (blue); few nuclei were stained with TUNEL in Figure 7A–G (red). In Figure 7H,I, antibodies against rabies and mumps are shown to have no effect, as most nuclei appear stained red with TUNEL.

**Figure 8:**
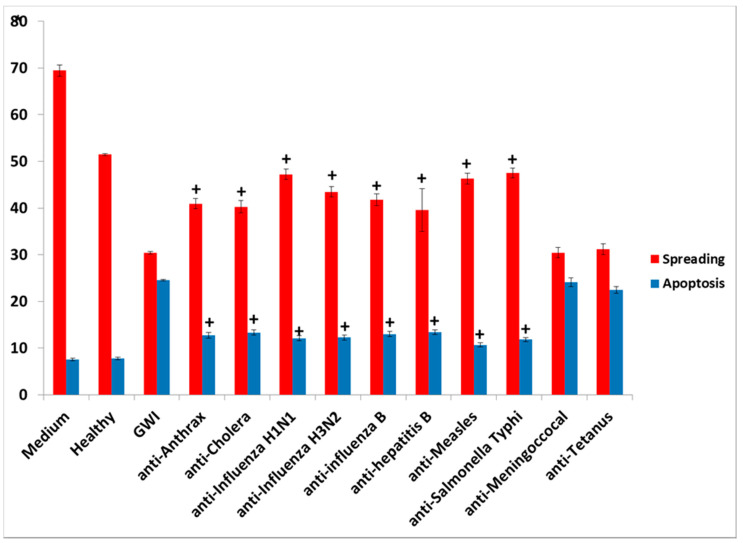
Combined spreading and apoptosis data analysis from all 15 GWI sera in cultured N2A cells; shown are the effects on N2A cells of GWI sera incubated in the presence of each of eight protective antibodies compared to GWI (**^+^***p* < 0.0001). Two examples are also shown of GWI sera incubated with anti-vaccine antibodies without a protective effect on cell spreading and apoptosis (anti-meningococcal, anti-tetanus), when compared to GWI.

Only a few of the used antibodies against the vaccines administered to GW veterans had a protective effect when co-incubated with GWI sera from 15 patients and then added to N2A cultures. These were antibodies to anthrax, cholera toxin, hepatitis B, hemagglutinin H1N1, H3N2, and B from influenza A and B strains, measles and *S. Typhi* polysaccharide Vi, which had a remarkable protective effect in both types of assays, as shown in the Figure 7 (examples of apoptosis with TUNEL assay) and 8 (cumulative effects of antibodies). The effects of antibodies to anthrax were examined in detail in a previous publication [17]. Nevertheless, none of the used antibodies had a total protective effect, since, in the control, healthy subject spreading was invariably more extensive (up to 55%) and apoptosis was less (5%–7%) compared to each one of the protective antibodies (Figure 8).

## 4. Discussion

We previously described a compromising effect of GWI serum on N2A cells, which, when exposed to this serum from different GWI subjects, underwent structural and functional changes. GWI serum induced a significant increase in neural network variability, indicating a harmful effect on neural network. Moreover, cells became aggregated, could not develop and/or retracted processes, and underwent apoptosis [12]. This effect could be due to a number of reasons, including toxic factors in the serum. In previous reports, it was demonstrated that GWI patients lacked six protective HLA alleles which are of paramount importance for the production of antibodies to various pathogens [8]. GWI veterans, in addition to having been potentially exposed to toxic environmental factors, were administered 20 different vaccines. Lack of protective HLA alleles might have prevented antibody formation to neutralize vaccine pathogens, thus leading to circulating pathogens as persistent pathogens [7]. The possibility then exists that persistent circulating pathogens had a compromising (harmful) effect on cultured neural cells.

In order to address this hypothesis, we examined the effect of vaccines administered to GW veterans which did not contain aluminum as adjuvant, since this chemical is toxic in cell cultures [25]. The vaccines tested were influenza strains A and B, typhoid, rabies, *Neisseria meningitis* (meningococcus), mumps–measles–rubella (MMR), varicella, and polio. In all cases, the lowest amount of vaccine having an effect was used in the experiments. From the tested vaccines, only influenza and *S. Typhi* had an adverse effect in culture, mimicking the effect of GWI serum, since their presence resulted in retraction of cell processes and/or inhibition of process formation, cell aggregation, and enhanced apoptosis.

The presence of influenza vaccine resulted in approximately a 40% decrease of spread cells, whereas *S. Typhi* led to a 75% reduction. Cell apoptosis was approximately enhanced 4× by both vaccines. This effect was due largely to the attenuated viruses or virus components contained in the vaccine, since co-incubation of antibodies directed against components of H1N1- and H3N2-like strains A of influenza with the vaccine restored cell spreading to a large extent, whereas antibodies to hemagglutinin of B strain had no effect. Apparently, the detrimental effect of the *influenza* vaccine on cell spreading was, thus, mostly due to the A strains.

Although the exact mechanism of damage of bacterial and viral pathogens on neural cells is not completely understood, several studies suggest that, in certain instances, binding of pathogen components on the cell membrane can lead to cell apoptosis. Pathogen–neural cell interactions could occur with inactivated or attenuated pathogens, as well as isolated capsular components of pathogens contained in vaccines. Thus, one or more of the hemagglutinin components of influenza strain H1N1, H3N2, and B, which the influenza vaccine used in our experiments contained, may have interacted with sialic acid residues [26,27,28] present on N2A cells [29]; these lectin components, following interaction with the cell membrane, caused cell aggregation [28]. Cell aggregation/agglutination was reported to increase membrane fragility [30]. Moreover, agglutinins were described to be cytotoxic, by binding to cell membranes and causing fusions [31] and collateral membrane damage [32]. A number of agglutinins were previously tested and found to be toxic to various cultured cell lines. The mechanisms of damage included impairing of multiple membrane transport systems [33], probably causing membrane destabilization [30] and creating pores [31,34] which, among other effects, may release K^+^ from the cell and create an electrolyte imbalance eventually triggering cell apoptosis [33]. Membrane changes resulting from pathogen–cell membrane interactions were reported to perturb cell function and eventually lead to the activation of apoptotic pathways/cell apoptosis, as observed with hepatitis B vaccine in mouse-lived hepatoma cell lines [25]. Apoptosis in these instances accompanies nuclear fragmentation and DNA release [27]. 

Similar mechanisms resulting in perturbed cell membranes could have occurred following the binding of *S. Typhi* polysaccharide Vi which was the active ingredient in the *S. Typhi* vaccine. The binding affinities of the influenza and *S. Typhi* vaccines with cell membranes could explain the observed toxicity. It is possible that bacterial and viral components of the vaccines without effects on cultured neural cells either did not bind or bound only with weak to moderate intensity to cell membranes, and they did not result in cell aggregation and/or membrane damage; pathogen toxicity dependent on the affinity for host cell membranes binding was reported in the case of different agglutinins tested for interactions with cell surfaces [27].

An explanation of the harmful effects of specific vaccines in neural cells would be the presence of vaccine pathogens in the serum of GWI patients, which led to the observed adverse effects on cultured N2A cells; these were similar to the effects of the influenza and *S. Typhi* vaccine. GWI serum, as well as each of these two vaccines, caused cell aggregation accompanied by inhibition of formation/retraction of cell processes and enhanced cell apoptosis. Moreover, co-incubation of healthy serum with the influenza vaccine did not result in a significant harmful effect in the cell spreading assay. A likely explanation for this finding was that the serum from a healthy veteran with two of the six protective alleles contained antibodies which effectively neutralized the harmful influenza A strains.

These observations lend support to the persistent antigen hypothesis [9]. It is possible that GWI patients lacking the protective HLA alleles were not able to form antibodies to one or more of the administered vaccines, allowing the pathogen to persist for prolonged time intervals [11] and resulting in long-term low-grade inflammation [7,12]. Indeed, persistent antigen was observed in immune CD4-deficient mice injected with a strain of influenza A virus, leading to lack of the appropriate immune response/lack of antibody formation [35]. Moreover, mice injected with iodinated human serum albumin (^125^I HSA) in order to examine the half-life of administered antigens were observed to maintain radioactivity in follicles of lymph nodes for extended time intervals [11]. 

The nature of persistent pathogens in the serum of GWI patients could, thus, lie, at least in part, in one or more or the administered vaccines, since at least two of those were observed to adversely affect structural and functional aspects of cultured N2A cells, mimicking the effect of GWI serum. The reason why only antibodies to anthrax, cholera toxin, hepatitis B, hemagglutinin H1N1, H3N2, and B from influenza A and B strains, measles, and *S. Typhi* polysaccharide Vi had a protective effect in N2A cultures is not clear, but could be due to variable toxicity of the antigenic components present in different vaccines. It is possible that one or more pathogens in these vaccines was more toxic compared to other vaccine components, whereby antibodies against them had no effect.

## 5. Conclusions

In summary, specific antibodies to specific GW-era vaccines exerted a substantial albeit partial protective effect when added to GWI-exposed N2A cells, allowing cell spreading and neuronal process formation and limiting the extent of apoptosis. The protective effect of antibodies was specific (only several of 20 antibodies tested had an effect), thus providing strong evidence for the hypothesis of persistent pathogens in GWI. The present study included 15 GWI patients who lacked the protective alleles; extending these approaches to a larger number of patients would strengthen the data reported herein. Moreover, additional laboratory and clinical approaches are required. For the latter, a pilot study with plasmapheresis in a small number of GWI patients is scheduled to address whether removal of persistent pathogens will result in alleviation of symptoms, which will also be accompanied by in vitro protective effects in N2A cells, similar to those described in this paper. Insofar as laboratory approaches are concerned, the identification of persistent pathogens in minimal amounts in the serum with Western approaches of the highest possible resolution could provide a more definitive answer; extensive proteomic approaches to detect minimal amounts of these pathogens could be used, among other methods. Thus, further studies are required in order to establish whether these in vitro observations may provide strategies for in vivo intervention with GWI.
